# Ebola Virus Disease in Children, Sierra Leone, 2014–2015

**DOI:** 10.3201/eid2210.160579

**Published:** 2016-10

**Authors:** Felicity Fitzgerald, Asad Naveed, Kevin Wing, Musa Gbessay, J.C.G. Ross, Francesco Checchi, Daniel Youkee, Mohammed Boie Jalloh, David Baion, Ayeshatu Mustapha, Hawanatu Jah, Sandra Lako, Shefali Oza, Sabah Boufkhed, Reynold Feury, Julia A. Bielicki, Diana M. Gibb, Nigel Klein, Foday Sahr, Shunmay Yeung

**Affiliations:** Save the Children, London, UK (F. Fitzgerald, J.C.G. Ross, F. Checchi);; University College of London Great Ormond Street Institute of Child Health, London (F. Fitzgerald, N. Klein);; Save the Children, Freetown, Sierra Leone (A. Naveed, M. Gbessay);; London School of Hygiene & Tropical Medicine, London (K. Wing, F. Checchi, S. Oza, S. Boufkhed, S. Yeung);; Kings Sierra Leone Partnership—Connaught Hospital, Freetown (D. Youkee);; Republic of Sierra Leone Armed Forces, Freetown (M.B. Jalloh, F. Sahr);; Ola During Children’s Hospital—Sierra Leone Ministry of Health, Freetown (D. Baion, A. Mustapha);; Cap Anamur (German Emergency Doctors)—Ola During Children’s Hospital, Freetown (H. Jah);; Welbodi Partnership—Ola During Children’s Hospital, Freetown (S. Lako);; Western Area Emergency Response Centre, Freetown (R. Feury);; St. George’s University of London, London, UK (J.A. Bielicki);; MRC Clinical Trials Unit at University College of London, London (J.A. Bielicki, D.M. Gibb)

**Keywords:** Ebola virus disease, children, Ebola, Sierra Leone, viruses

## Abstract

Little is known about potentially modifiable factors in Ebola virus disease in children. We undertook a retrospective cohort study of children <13 years old admitted to 11 Ebola holding units in the Western Area, Sierra Leone, during 2014–2015 to identify factors affecting outcome. Primary outcome was death or discharge after transfer to Ebola treatment centers. All 309 Ebola virus–positive children 2 days–12 years old were included; outcomes were available for 282 (91%). Case-fatality was 57%, and 55% of deaths occurred in Ebola holding units. Blood test results showed hypoglycemia and hepatic/renal dysfunction. Death occurred swiftly (median 3 days after admission) and was associated with younger age and diarrhea. Despite triangulation of information from multiple sources, data availability was limited, and we identified no modifiable factors substantially affecting death. In future Ebola virus disease epidemics, robust, rapid data collection is vital to determine effectiveness of interventions for children.

## Abstract 2

Children died rapidly, more than half in Ebola holding units before transfer to treatment units.

The Ebola virus disease (EVD) outbreak in West Africa during 2014–2016 comprised ≈28,600 cases and claimed ≈11,300 lives (*1*). The case-fatality rate (CFR) was high for Ebola virus (EBOV)–infected children <5 years of age (*2*–*4*). Outbreak conditions with overstretched health systems and paucity of data has meant there is little understanding of how modifiable clinical management, operational-, and response-specific factors might have affected outcomes of children with EVD (*3*,*5*).

During the outbreak, several different health service models for managing suspected EVD cases evolved. In the highly populated Western Area of Sierra Leone, including Freetown, on-site Ebola holding units (EHUs) were set up at health facilities. Their goal was to enable provision of normal healthcare to continue by screening patients before entry (*6*). Patients fulfilling screening criteria were admitted to the on-site EHUs for EVD testing. Persons testing positive for EBOV were transferred to Ebola treatment centers (ETCs). At the height of the outbreak, laboratory, ambulance, and bed capacities were overwhelmed, leading to substantial delays throughout the pathway, limited provision of clinical care, and long transfer distances (*7*,*8*). As the response scaled up, delays shortened and transfer distances decreased as more ETCs opened locally (*7*,*8*). These changing health systems factors possibly affected death and are amenable to modification in future outbreaks.

Patient data were collected on paper forms at EHUs and ETCs. Basic demographic and initial symptom information was telephoned to regional control centers for entry into electronic databases and transfer to international nongovernment organizations for epidemiologic surveillance (*3*,*5*,*9*,*10*). However, data on clinical management and key factors, such as caregiver accompaniment, were not included. Furthermore, communication between EHUs and ETCs was limited, meaning EHUs had no information about patient outcome after transfer, and data from ETCs were subject to substantial survivorship bias (*11*–*13*). Attempts to link clinical management data between sites were limited.

We describe the clinical features, management, and outcomes of children with EVD from initial presentation to final outcome. We also explore risk factors for death, in particular factors amenable to modification.

## Methods

### Study Population and Setting

**Figure 1:**
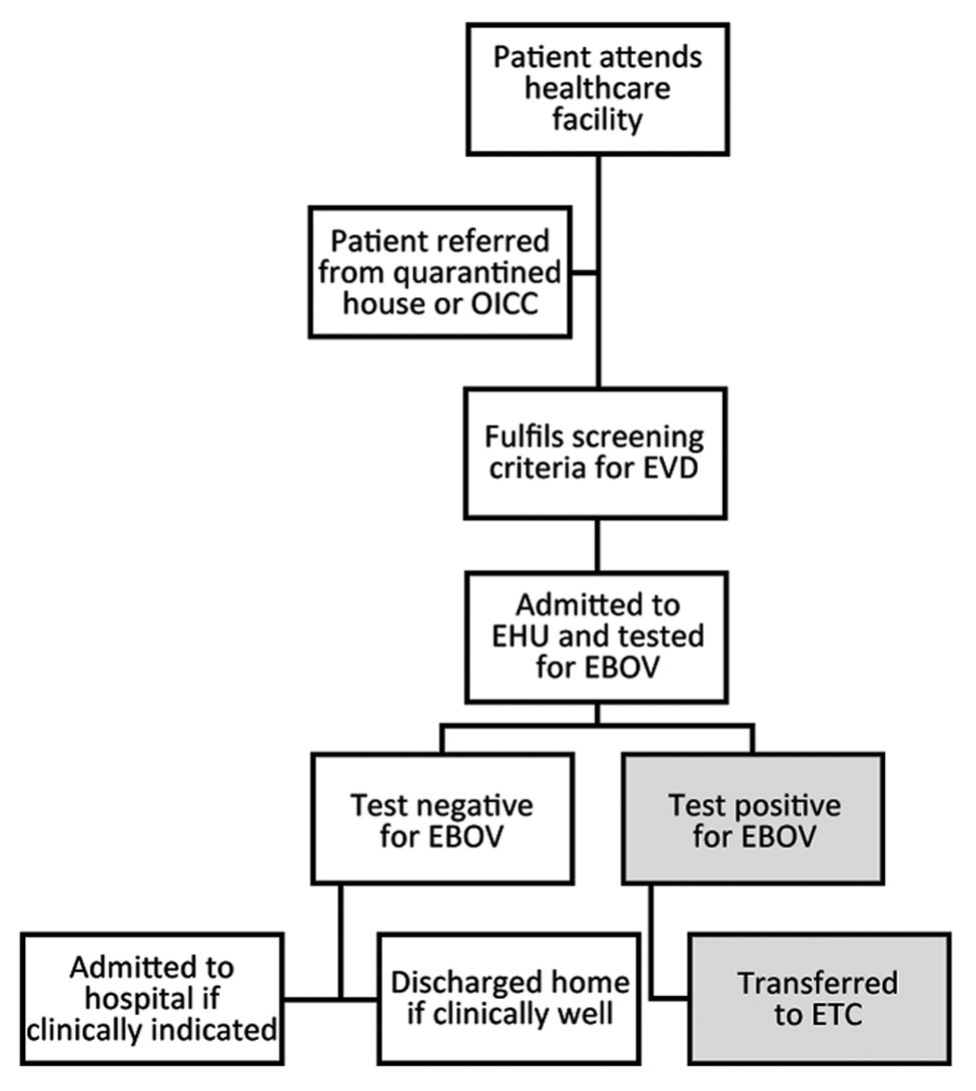
Patient care pathway of EHUs, Western Area (including Freetown), Sierra Leone, August 2014–March 2015. The OICC was set up to care for children with substantial Ebola virus exposure (usually a first-degree relative with confirmed EVD) and without relatives to care for them during the 21-day incubation period. Figure reproduced from (*8*) (Creative Commons License). EHU, Ebola holding unit; EVD, Ebola virus disease; OICC, observational interim care center.

All children <13 years of age admitted to 11 EHUs in the Western Area from August 14, 2014, through March 31, 2015, were eligible for inclusion, anticipating that the disease phenotype in adolescents might be similar to that in adults and that some factors (e.g., caregiver accompaniment) were more relevant for younger children (*3*,*14*). Patients were screened for EVD symptoms or contact with EBOV on entry to healthcare facilities. Patients whose signs and symptoms fulfilled the World Health Organization definition for a suspected case were admitted to on-site EHUs to have blood taken for EBOV PCR testing (Technical Appendix Figure) (*15*). Basic demographic data were reported to the Western Area Emergency Response Command Centre (WAERC). Samples were transported to specialist laboratories, which were usually off-site. Patients testing negative for EVD were discharged home or admitted to a routine (non-Ebola) hospital bed (Figure 1) (*8*). Patients testing positive were transferred to an ETC coordinated by the WAERC. Occasionally, if ETCs were full, a patient might stay at an EHU for the duration of illness and be discharged home directly.

Medical care varied among EHUs according to staffing capabilities and facilities available. The Sierra Leone Ministry of Health recommended that all patients admitted to an EHU receive antimalarial drugs and broad-spectrum antimicrobial therapy (*15*,*16*). Until late December 2014, most EHUs abstained from taking blood for tests other than EBOV PCR (WAERC, November 2014, unpub. data). Treatment was primarily supportive; some units operated a no-needle policy, providing only oral rehydration salt (ORS) solution and oral medications. Most units did not allow asymptomatic caregivers to enter an EHU high-risk area (i.e., Red Zone) with their children, because of the potential risk for nosocomial EBOV infection, so many children were admitted alone.

### Data Collection

**Figure 2:**
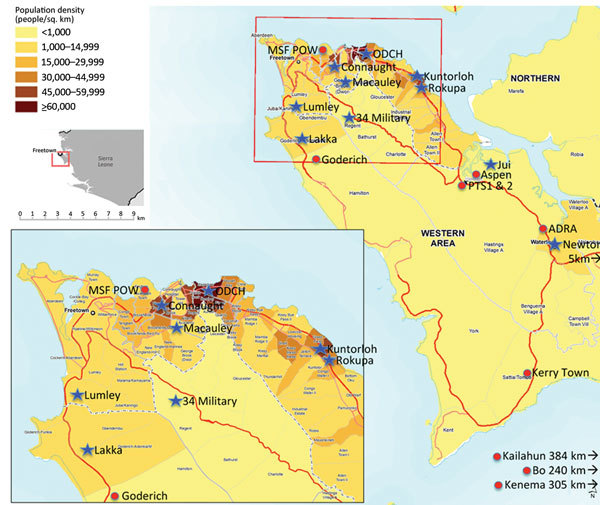
Location of Ebola holding units (blue star) and Ebola treatment centers (red circle), Western Area, Sierra Leone, January 2015. Population density map source: MapAction (cited 2015 Nov 8); reproduced with permission. Population figures are projected for 2014 from the 2004 census (http://www.mapaction.org/?option=com_mapcat&view=mapdetail&id=3589)..

Data stored electronically in the WAERC database included demographics and symptoms at presentation. To obtain more detailed data, we visited 11 EHUs, Ola During Children’s Hospital, and the Western Area ETCs (Figure 2) to extract data from paper records, including demographic data; contact histories; clinical features at and during admission; treatment received; laboratory results; and health system factors, such as duration of transfer to ETC and outcome (death or survival to discharge) (Technical Appendix Table 1). This information was supplemented by interviews with staff members, in particular about caregiver accompaniment, which was routinely documented only at 1 site. Information about outcomes after discharge was obtained from 2 survivor clinics in the Western Area and telephone calls to guardians. Data were cross-referenced with the WAERC database, test results from regional laboratories, child protection records, districtwide burial records, and the telephone service set up for community ambulance notification (Technical Appendix Table 1). We developed a scheme to ensure consistency in matching records from different sources (online Technical Appendix). Data were entered directly into a password-protected database (Epi Info version 7.1.4; Centers for Disease Control and Prevention, Atlanta, GA, USA). Personal identifiers were removed before analysis.

### Outcome and Potential Risk Factors

Possible outcomes were death (recorded at EHU or ETC) or survival (recorded as discharge from ETC or EHU). We considered variables encoding potential risk factors for outcome, including demographics (age, sex); duration from symptom onset to EHU arrival; symptoms at presentation; receipt of antimicrobial drugs, antimalarial drugs, or intravenous fluids at EHU; specific EHU attended; period in the epidemic (before or on/after January 9, 2015, when bed capacity first outstripped demand in the Western Area [*8*]); and whether the child was admitted alone, unaccompanied by a caregiver (online Technical Appendix).

We restricted a separate analysis to children transferred to ETCs. Variables were days from EHU admission to ETC transfer, transfer distance, ETC attended, and receipt of medications or intravenous fluids at the ETC. If health information was available for survivors after discharge, it was collected but was not a primary focus of this study.

### Sample Size

At study conception in December 2014, we estimated 300 children would have sought care in Western Area EHUs. We aimed to obtain data on all these children (see calculations in online Technical Appendix).

### Statistical Analysis and Ethical Considerations

We conducted a descriptive analysis on all children admitted to EHUs. Among children with known outcome, we conducted univariable and multivariable analyses to explore factors potentially affecting outcome (Technical Appendix). We accounted for missing data with multiple imputation using chained equations (Technical Appendix) (*17*). All analyses were conducted by using Stata version 14.0 (StataCorp LP, College Station, TX, USA). We obtained approval from the Sierra Leone Ethics and Scientific Review Committee and the London School of Hygiene and Tropical Medicine Ethics committee (ref. 8924).

## Results

### Overall Outcomes

**Figure 3:**
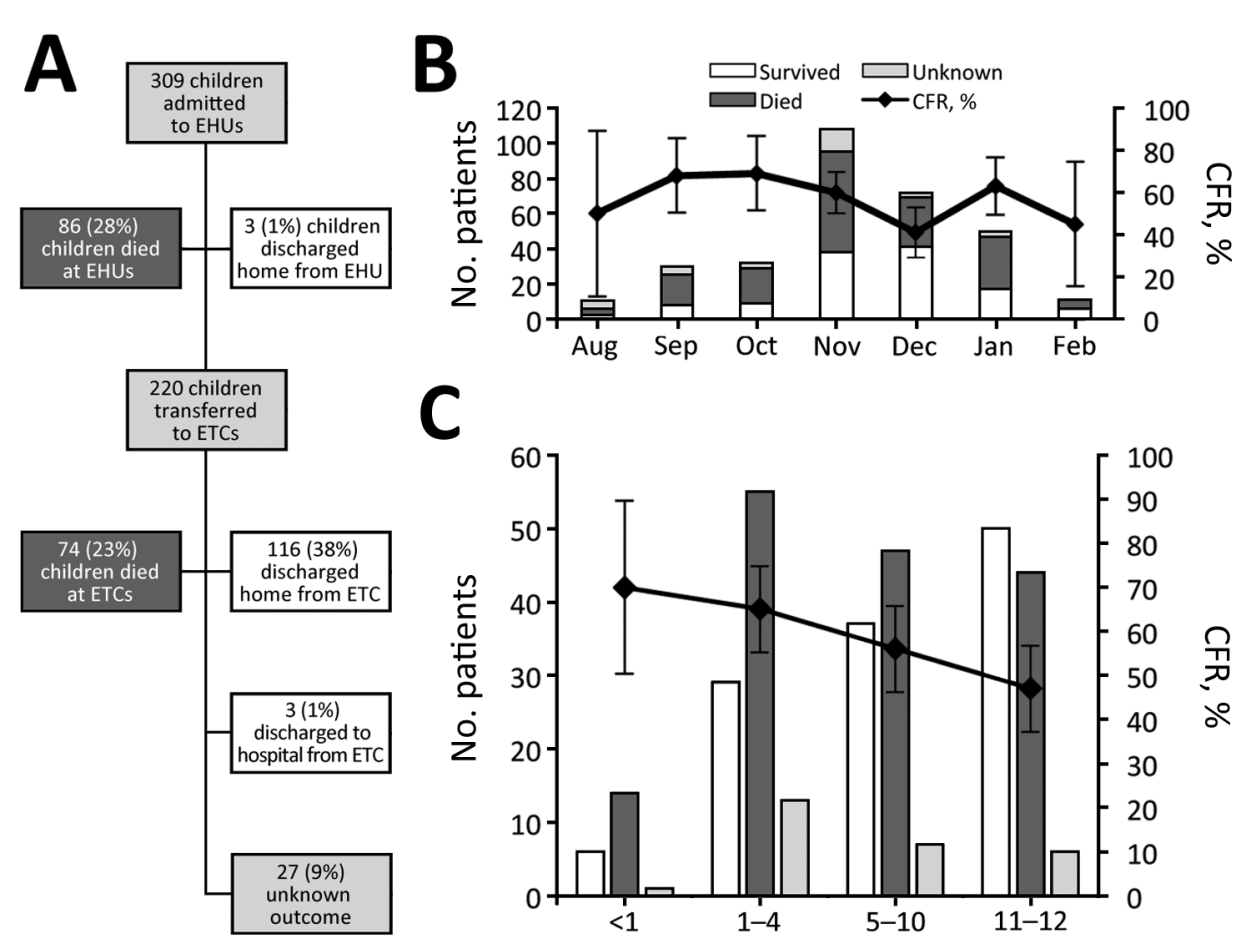
Outcome characteristics of children <13 years of age with Ebola virus disease, Western Area, Sierra Leone, August 2014–March 2015. A) Patient outcome flowchart; B) patient count and case-fatality rate (CFR) by month; C) Patient count and CFR by age. Error bars indicate 95% CIs.

Our study comprised all 309 children 2 days–12 years of age admitted to EHUs and testing positive for EBOV (median age 6 years, interquartile range [IQR] 3–10 years; 158 [51%] female). Outcome (death or discharge) was available for 282 (91%) children; CFR was 57% (95% CI 51%–63%) (Table 1). Eighty-six (28%) children died at EHUs, and 223 (72%) were transferred to ETCs, where an additional 74 (24%) died. Therefore, 55% of deaths occurred at EHUs and 45% at ETCs (Figure 3, panel A). Of children transferred to ETCs, 116 (38%) were discharged home and 3 (1%) were discharged to a hospital (Figure 3, panel A). Three children were discharged home from EHUs without transfer. Median duration of EHU/ETC admission among children who died was 3 days (IQR 1–5 days) and for survivors was 17 days (IQR 11–20 days). Outcomes were missing for 27 (9%) children, all of whom had been transferred out of EHUs to ETCs (Figure 3, panel A). The CFR was highest in September and October 2014, a period of intense EBOV transmission, and peaked again in January 2015 (Figure 3, panel B).

**Table 1:** Descriptive, univariable, and multivariable analysis of 282 Ebola-positive children who attended an EHU and for whom outcome was recorded, Western Area, Sierra Leone, August 2014–March 2015*

Variable	Total, no. (%)	Survived, no. (%)	Died, no. (%)	Crude OR† (95% CI)	Multivariable adjusted OR‡
Total§	282 (100)	122 (43)	160 (57)	–	–
Sex					
F	146 (52)	70 (48)	76 (52)	1	1
M	136 (48)	52 (38)	84 (62)	1.49 (0.93–2.39¶	1.42 (0.86–2.36)
Age, y					
Mean (SD)	6.6 (3.9)	7.4 (3.6)	6.0 (4.0)	OR per +1 y	
Median (IQR)	7 (3–10)	8 (4–10)	5 (2–10)	0.91 (0.85–0.97)	0.92 (0.86–0.98)
Age group, y					
5 to <12	178 (63)	87 (49)	91 (51)	1	–
0 to <5	104 (37)	35 (34)	69 (66)	1.88 (1.14–3.11)¶	–
Days from symptom onset to EHU presentation					
Mean (SD)	3.6 (3.0)	3.6 (3.1)	3.5 (3.0)	1.16 (0.59–2.29)	–
Median (IQR)	3 (2–4)	3 (2–4)	3 (2–4)	OR per +1 d	
Signs and symptoms					
Fever,# n = 204					
No	9 (4)	6 (67)	3 (33)	1**	–
Yes	195 (96)	83 (43)	112 (57)	2.70 (0.66–11.11)	–
Fatigue/weakness, n = 163					
No	4 (2)	1 (25)	3 (75)	1**^5^**	–
Yes	159 (98)	65 (41)	94 (59)	0.48 (0.05–4.74)	–
Common symptoms, n = 160					
Fever or fatigue	8 (5)	3 (37)	5 (63)	1	–
Both	152 (95)	63 (41)	89 (59)	1.07 (0.58–1.97)	–
Vomiting/nausea, n = 205					
No	83 (40)	37 (45)	46 (55)	1	–
Yes	122 (60)	51 (42)	71 (58)	1.13 (0.64–2.00)	–
Diarrhea, n = 198					
No	108 (55)	56 (52)	52 (48)	1	1
Yes	90 (45)	31 (34)	59 (66)	1.94 (1.11–3.39)¶	1.91 (1.08–3.39)
Anorexia, n = 201					
No	44 (22)	21 (48)	23 (52)	1	–
Yes	157 (78)	64 (41)	93 (59)	1.30 (0.66–2.55)	–
Abdominal pain, n = 188					
No	78 (41)	32 (41)	46 (59)	1	–
Yes	110 (59)	51 (46)	59 (54)	0.82 (0.49–1.4)	–
Hiccups,†† n = 188					
No	179 (95)	79 (44)	100 (56)	1	–
Yes	9 (5)	3 (33)	6 (67)	1.58 (0.38–6.52)††	–
Difficulty swallowing, n = 191					
No	147 (77)	64 (44)	83 (56)	1	–
Yes	44 (23)	19 (43)	25 (57)	0.98 (0.44–2.17)	–
Difficulty breathing, n = 190					
No	164 (86)	72 (44)	92 (56)	1	–
Yes	26 (14)	8 (31)	18 (69)	1.74 (0.69–4.55)	–
Muscle pain, n = 185					
No	104 (56)	45 (43)	59 (57)	1	–
Yes	81 (44)	37 (46)	44 (54)	0.93 (0.53–1.64)	–
Joint pain, n = 186					
No	99 (53)	44 (44)	55 (56)	1	–
Yes	87 (47)	39 (45)	48 (55)	1.00 (0.57–1.77)	–
Headache, n = 185					
No	77 (42)	29 (38)	48 (62)	1	1
Yes	108 (58)	53 (49)	55 (51)	0.64 (0.34–1.19)¶	0.60 (0.32–1.13)
Conjunctivitis, n = 190					
No	118 (62)	54 (46)	64 (54)	1	–
Yes	72 (38)	30 (42)	42 (58)	1.18 (0.63–1.90)	–
Rash, missing n = 190					
No	181 (95)	79 (44)	102 (56)	1	–
Yes	9 (5)	3 (33)	6 (67)	1.55 (0.38–6.39)††	–
Unexplained bleeding,†† n = 192					
No	189 (98)	81 (43)	108 (57)	1	–
Yes	3 (2)	1 (33)	2 (67)	1.50 (0.13–16.83)††	–
Rare symptoms,†† n = 189					
No	169 (89)	75 (44)	94 (56)	1	–
Yes	20 (11)	7 (35)	13 (65)	2.04 (0.84–4.97)¶	–
Malaria positive by RDT, n = 16					
No	14 (88)	9 (64)	5 (36)	–‡‡	–
Yes	2 (13)	0	2 (100)	–	–
Admitted accompanied, n = 181					
No	69 (38)	32 (46)	37 (54)	1	–
Yes	112 (62)	44 (39)	68 (61)	1.22 (0.65–2.28)	–
Date of presentation, n = 282					
Before Jan 9	256 (91)	114 (45)	142 (55)	1	–
On/after Jan 9	26 (9)	8 (31)	18 (69)	1.81 (0.76–4.31)¶	–
Common medications,§§ n = 149					
Antimicrobial or antimalarial drug at EHU	22 (15)	7 (32)	15 (68)	1	–
Antimicrobial and antimalarial drug at EHU	127 (85)	53 (42)	74 (58)	0.64 (0.32–.30)	–
Intravenous fluids at EHU					
No	270 (96)	117 (43)	153 (57)	1	–
Yes	12 (4)	5 (42)	7 (58)	1.07 (0.33–3.46)	–
EHU					
Ola During Children’s Hospital	112 (40)	41 (37)	71 (63)	1	–
Connaught	57 (20)	20 (35)	37 (65)	1.06 (0.55–2.08)	–
Lumley	13 (5)	7 (54)	6 (46)	0.49 (0.16–1.57)	–
Rokupa	24 (9)	15 (63)	9 (38)	0.35 (0.14–0.86)	–
Macauley	16 (6)	5 (31)	11 (69)	1.27 (0.41–3.91)	–
Newton	20 (7)	10 (50)	10 (50)	0.58 (0.22–1.50)	–
Kerry Town Suspect Ward	5 (2)	3 (60)	2 (40)	0.38 (0.06–2.40)	–
Police Training Schools 1 and 2	14 (5)	9 (64)	5 (36)	0.32 (0.10–1.02)	–
34 Military Hospital	10 (4)	5 (50)	5 (50)	0.58 (0.16–2.11)	–
Aspen	4 (1)	2 (50)	2 (50)	0.58 (0.08–4.36)	–
Kuntorloh	1 (0)	1 (100)	0	–	–
Jui	6 (2)	4 (67)	2 (33)	0.29 (0.05–1.65)	–

*EHU, Ebola holding unit; IQR, interquartile range; OR, odds ratio; –, omission of variables according to p value as described in the footnotes that follow. †Multiple imputation used to account for missing data for all estimates with missing data. Multiple imputation model included all variables in this table with missing data (unless specified) plus the complete variables: outcome status, sex, age, date of admission, and EHU. For comparison of multiple imputation with a complete records analysis, see Technical Appendix Table 2. ‡Multivariable regression model included age, sex, diarrhea, and headache (selected for inclusion using a backward stepwise approach) with multiple imputation applied as explained in the previous footnote. §Total children admitted to EHUs for whom complete outcome information was available. ¶p<0.20 (Wald test); therefore, variable was included in an initial multivariable model from which the final multivariable presented in this table was obtained through a backward stepwise approach (online Technical Appendix, “Statistical Analysis” section and Technical Appendix Table 2). #All symptoms in this table: recorded upon presentation at EHU. **Fever and fatigue/weakness: insufficient numbers of children recorded as “No” for these variables to include in multiple imputation model; therefore, the OR for a complete records analysis is shown here. A “common symptoms” variable was created to enable inclusion of data related to these symptoms in multiple imputation and multivariable regression models. ††Rash, hiccups, and unexplained bleeding: insufficient numbers of children recorded as “Yes” for these variables to include in multiple imputation model. A “rare symptoms” variable was created to enable inclusion of data related to these symptoms in multiple imputation and multivariable regression models. ‡‡Insufficient numbers for regression analysis. §§Combined variable created because of the low numbers of children recorded as not having received either medication.

### Clinical Features

Data were available on symptoms at presentation for ≈70% of children (Table 1). Fever was the most prevalent symptom (99%), followed by fatigue/weakness (80%), anorexia (79%), vomiting (59%), and diarrhea (44%); unexplained bleeding was rare (1%). Median reported duration of symptoms before presentation was 3 days (IQR 2–4 days). Of 193 children for whom exposure data were available, 151 (78%) had documented prior contact with a person with EVD, and 42 (22%) had documentation of no prior exposure to EVD.

Additional clinical information was available for a subset of 88 patients for the duration of EHU/ETC admission. In addition to 3 children with spontaneous bleeding at presentation, 7 (8%) had bleeding after admission. In order of frequency, manifestations were bleeding gums, epistaxis, hematemesis, melena, and vaginal bleeding. Only 1 of these 10 children survived to discharge. Seizures were recorded during admission in 6 (7%) children: 3 died, 2 were discharged in a comatose state, and 1 recovered with no reported sequelae.

### Laboratory Features

Additional blood test results were available for 36 children from 3 health facilities (Table 2). These children were of similar age (median 6 years, IQR 3–7 years) to children in the overall cohort, but the CFR was lower (11/36 [31%; 95% CI 16%–48%]). Children who died were younger than children who survived (data not shown).

**Table 2:** Blood test results for children attending Ebola holding units and Ebola treatment units for whom blood test results were available, Western Area, Sierra Leone, August 2014–March 2015*

Laboratory value (reference)	Median value among patients who died	Median value among patients who survived	p value†
Leukocyte count (4–11), × 10^9^/L	33, n = 7	9.2, n = 22	0.067
Lymphocyte count (1–3.2), × 10^9^/L	6.1, n = 7	2.9, n = 21	0.007
Granulocyte count (2.5–7.5), × 10^9^/L	19.9, n = 7	4.9, n = 22	0.009
Hemoglobin (135–175), g/L	123, n = 7	110, n = 22	0.097
Platelets (150–430), × 10/L	376, n = 7	179, m = 22	0.17
Sodium (128–145), mmol/L	127, n = 10	131, n = 24	0.11
Potassium (3.6–5.1), mmol/L	3.9, n = 7	4.1, n = 23	0.9
Urea (2.5–7.9), mmol/L	16.2, n = 11	4.2, n = 24	<0.001
Creatinine (53–106), mmol/L	120, n = 10	49, n = 24	0.003
Albumin (33–50), g/L	26, n = 11	31, n = 20	0.25
Aspartate transaminase (11–35), U/L	2,000, n = 8	159, n = 17	0.001
Alanine transaminase (10–48), U/L	667, n = 11	131, n = 18	<0.001
Creatine kinase (39–380), U/L	2,544, n = 8	623, n = 18	0.13
C-reactive Protein (0–7.5), mg/L	96, n = 10	8, n = 17	0.007
Viral cycle threshold, range‡	14.3–35.8, n = 40	17–38.2, n = 42	NA

*n values indicate number of patients in category for whom value was available. NA, not applicable †Wilcoxon rank-sum test used because of small sample size. ‡Different reference ranges used.

Tests were taken a median of 3 days (range 0–18 days) after admission at an EHU. Leukocyte count (predominantly granulocytes) and C-reactive protein were elevated, and there was considerable renal and liver function derangement, particularly among children who died. Hyponatremia occurred among children who survived and who died, but potassium levels tended to be within normal limits. Hypoglycemia (blood glucose <4.0 mmol/L [reference 4–6.9 mmol/L) was common among children who died (55% [95% CI 23%–83%]) and who survived (30% [95% CI 13%–53%]).

Cycle threshold (C_t_) represented the point at which a quantitative PCR was interpreted as positive: the lower the C_t_, the higher the initial viral load. Results were available from 90 children (42 who survived; 40 who died; 8 unknown; median age [IQR] 8.5 years [4–11 years], 5.0 years [3–10 years], and 6.0 years [2.9–10.5 years], respectively). The range of C_t_ was similar for children who died and who survived (14.3–35.8 vs. 17.0–38.2) (Table 2).

### Health Systems and Clinical Management Factors

Median duration of admission at EHU before transfer or death was 2 days (IQR 1–3 days). Outcomes were available for 193 children who survived to transfer to an ETC. Twenty-five (13%) were transferred directly onto an on-site treatment ward, but most (168 [87%]) traveled 5–380 km (median 25 km [IQR 19.5–45.0 km]). For 201 children for whom information was available about caregiver accompaniment (written documentation in 119 [59%], information from staff interviews alone in 84 [41%]), 74 (37%) were documented as unaccompanied admissions.

Treatment was primarily supportive but ranged from aggressive intravenous or intraosseous fluid resuscitation with laboratory monitoring (more common in ETCs) to ORS and oral medications. Medications received at either EHUs or ETCs were recorded for 178 (58%) children: 99% received antimicrobial drugs and ORS, 85% antimalarial drugs, and 19% intravenous fluids.

### Risk Factors for Death

In univariable analysis, younger age (odds ratio [OR] per year of life 0.91 [95% CI 0.85–0.97]) and diarrhea at presentation (OR 1.94 [95% CI 1.11–3.39]) were significantly associated with death. CFR was highest for infants (70%) (Figure 3, panel C).

In multivariable analysis, age was the strongest predictor of death (adjusted OR 0.92 [95% CI 0.86–0.98] per 1-year increment in age) (Table 1; Figure 3, panel C). Diarrhea at presentation was associated with death (OR 1.91 [95% CI 1.08–3.39]). None of the following were associated with death: time from symptom onset to EHU admission, receipt of specific medications or intravenous fluids, attendance at any particular EHU, or being accompanied by a caregiver (Table 1; Technical Appendix Table 2). We found minimal difference between estimates obtained by analyzing only complete records compared with accounting for missing data using multiple imputation (Technical Appendix Tables 3, 4).

For 193 children transferred to ETCs for whom outcomes were known (Technical Appendix Tables 5, 6), neither longer duration of EHU stay before transfer nor longer transfer distances appeared detrimental. Outcomes between ETCs varied considerably, but CIs were wide. Receipt of medications or intravenous fluids at the ETC also were not associated with survival.

### Health Status of Survivors

Of 122 surviving children, data from survivor clinics or telephone interviews were available for 42 (34%). Twenty-five (60%) reported no problems; 6 (14%) were referred for ophthalmologic review with possible uveitis, 1 with monocular blindness; 3 (7%) had hearing problems; 2 (5%) had alopecia; 2 (5%) had joint pains; and 1 (2%) depressed affect. Of 2 children discharged without fully recovering consciousness, 1 was recovering and being cared for at home; the second had made little improvement in hospital.

## Discussion

We aimed to identify modifiable operational and clinical management factors that could affect the outcome of EVD in children. The relative completeness of outcome data (91%) was possible only through a high level of collaboration between government and nongovernment organizations, triangulating available data sources. This collaboration enabled crucial matching of clinical management data between EHUs and ETCs, which had not been undertaken previously. That 55% of deaths occurred in EHUs highlights the importance of data pooling. Data from ETCs alone need to be interpreted in the context of substantial survivorship bias (*11*,*12*,*18*). Patients spent a median of 2 days at an EHU before ETC transfer, a critical period given the rapid progression to death.

Cohorts of children reported from previous outbreaks have been smaller, 1 comprising 20 laboratory-confirmed cases and another comprising 55 patients <22 years of age (*4*,*19*). The largest study of children from this outbreak was an international cohort documenting epidemiologic findings from across the 3 most affected countries based on data reported to regional control centers (*3*). Although the sample size included was much larger (2,991 confirmed or probable cases in patients <16 years of age), outcomes were available for 42%–59% (varying with age), versus 91% in our study. Furthermore, data were not available about clinical management or caregiver accompaniment.

Our study concurs with previous analyses reporting that young age, particularly infancy, is a risk factor for death from EVD (*2*,*3*,*20*–*23*). Progression to death was swift (median time 3 days from admission [IQR 1–5 days]), more rapid than reported in mixed age cohorts, and the overall death rate was high (*2*,*9*). Compared with the international cohort data from this outbreak, the children in this cohort progressed more rapidly to death. The shortest mean duration from admission to death in the international cohort was 3.7 days for children 1–4 years of age and in other age groups was longer (*3*). Gastrointestinal symptoms predominated here as in mixed age cohorts, although diarrhea was less common in our study (45%) than in the international cohort study (60%) (*5*,*13*,*24*). Diarrhea at presentation, most likely a proxy for more severe disease, appeared to nearly double the risk for death. Few other clinical features at presentation appeared to be associated with death, possibly because of missing or unreliable data; in particular because so many children were unaccompanied, contact history, history of symptoms before attendance, and symptom duration must be cautiously interpreted. Contact history was denied in 42 (22%) children, implying a potential incentive for concealment, which further highlights the need for caution in interpreting these data. This study was powered to detect effects with an OR of ≈2, and the wide CIs for most risk factors mean that real associations not detected by this study cannot be ruled out.

Hypoglycemia in children with EVD has been assumed but not previously demonstrated (*15*). In this cohort, hypoglycemia was frequent and severe (40% of children tested had blood sugar <4.0 mmol/L). Hypoglycemia should be actively sought and treated as a priority, and dextrose should be included in maintenance intravenous fluids to minimize risk for hypoglycemia if monitoring is unavailable. The blood tests in this study were taken at the point of cannulation with no prior intravenous fluids received. As reported in a previous mixed-age cohort (*12*), other laboratory features include a dramatically raised leukocyte count (predominantly neutrophils) and derangement of renal and liver function. These tests were conducted a median of 3 days after EHU admission and thus are likely to represent a less severe phenotype of disease than in those who died more rapidly. Data from 12 children <6 years of age in the Guinea JIKI trial also demonstrated raised serum creatinine, creatine kinase, and liver function enzymes, although less frequently than in our study (*25*). In contrast, a study from Gulu, Uganda, found no relation between serum chemistry results and death, although an association was seen with markers of immune activation (*19*). A high viral load (low C_t_ on PCR) has been demonstrated to be associated with death, but because our study was multicenter, the tests were conducted in different laboratories using different assays, making comparisons inappropriate (*25*–*28*).

In terms of clinical management factors, we found no evidence that intravenous fluids had a protective effect on survival, although our study lacked power for this analysis. A case series of adults and children in Liberia also found that intravenous fluids were not protective (*29*). This finding may reflect sicker patients being prioritized for fluid resuscitation. However, aggressive fluid resuscitation in the context of limited monitoring (as was the case in most EHUs) may also have entailed risks. Further research into the safest methods of fluid resuscitation and effective fluid balance monitoring in the Red Zone are crucial, alongside accurate documentation of caregiving.

Nearly 40% of children, even infants, were admitted unaccompanied to EHUs. Unaccompanied children are vulnerable to inadequate oral fluid resuscitation and the hazards of high-concentration chlorine on tap or in basins before even considering the emotional trauma of separation (*30*,*31*). Furthermore, such children could contribute to nosocomial EBOV transmission because they are difficult to keep in their bed space. The heat of personal protective equipment meant that healthcare workers could not spend long within the Red Zone supervising children (*30*,*31*). Many units had started to have survivors work in Red Zones because they are understood to be at vanishingly low risk for re-infection (*32*,*33*). This option could be explored in future outbreaks to ensure adequate care for children.

The overwhelming majority (87%) of children who survived long enough to be moved from an EHU were transferred at least 5 km. Even unaccompanied children and young infants faced transfers up to 380 km to the nearest available ETC bed in very basic ambulances. All of the children in this study who had unknown outcomes were transferred out of EHUs and might have been lost to their families in addition to epidemiologic follow up. Prioritizing transferring children to locations close to their homes could minimize the chance of this loss. Although in our study, neither caregiver presence nor transfer distance was significantly associated with outcome, in the interests of the child both issues could be planned for in future outbreaks.

Although our study has outcomes for 91% of children, missing data and data reliability are a limitation. Transferring information, such as patient observations and medications given in the Red Zone to the low-risk Green Zone where notes were kept, was challenging. Solutions varied from scanners or radios to shouting over the fence, but none were ideal. Stethoscopes were banned, and even simple equipment, such as weighing scales and clocks, had a limited lifespan because of the high concentrations of chlorine used for cleaning. Some analyses lacked power, with wide CIs for some risk factors. Pooling data in this study with other similar cohorts could help identify additional risk factors for survival.

Children have previously been relatively spared by EVD (*20*,*34*,*35*). As the West Africa epidemic progressed, children constituted an increasing proportion of EVD cases (*3*,*36*,*37*). It is possible that earlier in the outbreak, more children were dying unreported at home, given that before the EVD epidemic, Sierra Leone had one of the highest infant mortality rates in the world (*38*). Thus, data presented here also might be subject to survivorship bias. A change in health-seeking behavior, namely bringing very sick children to hospital, might have contributed to the second peak in CFR later in the outbreak (Figure 3, panel B). Documented duration of symptoms before attendance did not change over time (data not shown) but might have been unreliably recorded.

The overarching messages of our study are 3-fold. First, death rates in children were high, and children died even more rapidly than previously documented (*3*). Second is the apparent lack of association between death and potentially modifiable factors that could alter outcome after infection. This lack of association calls for urgent prioritization of interventions targeted to prevent EVD in children. Children have been neglected thus far in EVD vaccine development, and this knowledge gap should be addressed (*39*,*40*). Furthermore, children should be included as a priority in future clinical trials of supportive care methods and of antiviral drugs. Third, the paucity and quality of data and the lack of studies pooling clinical management data from different sites need to be tackled. Many records were already lost when this study was conducted; any available data need to be urgently salvaged and shared. Plans for safe, rapid, and accurate data collection must be prioritized in outbreak planning to identify simple interventions that might improve outcome. We should act or miss a vital opportunity to learn how better to combat EVD in future epidemics.

Technical Appendix Additional methods and details for study of children <13 years of age with Ebola virus disease, Western Area, Sierra Leone, August 2014–March 2015
