# Supplementary material for: Ebola Virus Disease in Children, Sierra Leone, 2014–2015
Source: Emerg Infect Dis. 2016 Oct;22(10):1769–77. doi: 10.3201/eid2210.160579 (PMC5038433; doi:10.3201/eid2210.160579)
Supplement: Technical Appendix — Additional methods and details for study of children <13 years of age with Ebola virus disease, Western Area, Sierra Leone, August 2014–March 2015 [file 16-0579-Techapp-s1.pdf]

Article DOI: http://dx.doi.org/10.3201/eid2210.160579

Ebola Virus Disease in Children, Sierra Leone, 2014–2015

Technical Appendix

Scheme for Matching Patient Data Across Different Locations

To ensure patients were accurately traced across different locations (i.e., from Ebola holding centers [EHUs] to Ebola treatment centers, the Western Area Emergency Response Command Centre database, burials database, and laboratory results database) a scheme was developed to ensure consistency in matching.

A complete match consisted of the criteria below:

1. Matching Western Urban/Rural Area number and matching name. This number was allocated with each case investigation form but was used inconsistently;
2. Matching name, age, and case investigation form date;
3. Four or more of name, age, case investigation form date, address, EHU, eventual status (positive/negative/transferred/discharged);

A partial match consisted of ≥3 of name, age, case investigation form date, address, EHU, eventual status (positive/negative/transferred/discharged). Small discrepancies in name spelling (e.g., Mohammed and Mohamed) could still be included as a complete match, but larger discrepancies of several letters (e.g., Abu and Abubakar) were a partial match. Matching was performed by 2 investigators. Any discrepancies between the 2 investigators' categorization were raised with the lead investigator (F.F.) with whom the final decision rested. Partial matches were reviewed by the lead investigator and either discarded or included depending on any additional information available (e.g., from telephone follow-ups). All complete matches were included in the analysis.

Sample Size

As discussed in the main text, at outset we estimated that 300 children would have sought care at EHUs in the Western Area. Assuming a case-fatality rate of 50% in the unexposed group for any particular risk factor, we estimated this sample size was sufficient to detect an effect size (odds ratio [OR]) of 2.0 (with an α of 0.05).

Statistical Analysis

In addition to that described in the Methods, numerically rare or ubiquitous symptoms and treatments were grouped to enable analysis (fever and fatigue into a "common symptoms" variable; skin rash, bleeding, and hiccups into a "rare symptoms" variable; and antimicrobial and antimalarial drugs into a "common medications" variable). Other date of admission variables considered included a categorical month of admission variable (8 categories) and a categorical 2-month of admission variable (4 categories), but low numbers in each category limited the usefulness of these variables.

Risk factor analysis was then conducted on a cohort restricted to children for whom outcome was known. Initial analysis involved tabulation of results by outcome status (died vs. survived), followed by univariable and multivariable logistic regression to identify variables associated with death. Although in theory it would have been possible to perform time-to-event analysis (enabling analysis of whether, for example, receipt of medication prolonged life), we decided to analyze death as a binary variable because 1) death was high and disease duration (follow-up time) short and 2) the date of death was not recorded for 15% of children who died (meaning that multivariable time-to-event analysis would have required an additional imputation step compared with analysis using logistic regression). We checked our assumption that a time-to-event analysis would produce similar results by restricting the cohort to those with known outcome dates and performing a Cox regression analysis, and comparing the crude hazard ratios (HRs) with the ORs obtained from a logistic regression analysis of the same restricted cohort. There was minimal difference in results obtained, and no alternative conclusions would have been made, e.g., HRs for age <5 years versus ≥5 years was 1.59 (95% CI 1.13–2.24), compared with an OR of 1.80 (95% CI 1.05–3.07), HR for number of days from symptom onset to admission (HR per +1 day) was 1.00 (95% CI 0.93–1.07), compared with an OR of 0.99 (95%

CI 0.89–1.10), and HR for receiving both common medications versus only 1 medication was 0.73 (95% CI 0.40–1.34), compared with an OR of 0.57 (95% CI 0.20–1.62). For multivariable analysis, an initial model was fitted that included only variables with a (Wald test) p value of <0.20 in univariable analysis. A backward stepwise approach was then applied to obtain a final model, removing variables with p>0.10 one by one. Given the relatively small size of the dataset and the number of possible adjustments in multivariable analysis, we chose a p value cutoff of 0.1 (rather than 0.05), so that variables only weakly associated with the outcome in our dataset would remain in our final mode (to avoid a possible type II error due to the size of the dataset). This model was presented in the main results tables, with 2 additional models included in Technical Appendix Table 2 for reference: 1 model including all variables with p<0.20 in univariable and another model that included all variables available for analysis (a fully adjusted model). This process was repeated for the secondary analysis restricted to children entering the Ebola treatment center (with the alternative models for this analysis presented in Technical Appendix Table 6). Missing data were assumed to be missing at random (*1*), and were accounted for using multiple imputation by chained equations. All available variables were included in the multiple imputation model, and we created 10 imputed datasets, which were combined for analysis. Our missing-at-random assumption was based on a priori reasoning that missing data across all variables would be dependent on other recorded variables. EHU admitted to and time period within the epidemic were considered as likely to be of particular importance because of differences in patient and data handling approaches between EHUs and over time. A tabulation was performed to assess this by looking at the association between having no symptoms recorded (vs. at least 1 symptom recorded) and several key variables with data fully recorded (Technical Appendix Table 3). A complete-records sensitivity analysis was also performed, to enable comparison with the results obtained by using multiple imputation (Technical Appendix Table 4).

References

1. Sterne JA, White IR, Carlin JB, Spratt M, Royston P, Kenward MG, et al. Multiple imputation for missing data in epidemiological and clinical research: potential and pitfalls. BMJ. 2009;338:b2393. PubMed http://dx.doi.org/10.1136/bmj.b2393

2. World Health Organization. Clinical management of patients in the Ebola treatment centres and other care centres in Sierra Leone: a pocket guide. Interim emergency guidelines. Sierra Leone adaptation. Geneva: The Organization; 2014

Technical Appendix Table 1. Data sources*

Site	Data source	Data collected
EHU	Case investigation form	Demographics (age, address, vital status); date of symptom onset; date of admission to EHU; symptoms up until presentation; contact history; date of death or discharge if occurs from EHU
EHU	Site-specific clinical records and drug charts	Ebola PCR result; vital signs; symptom progression; receipt of enteral or parenteral fluids; medications given; laboratory results
EHU	Admission book	Whether accompanied by caregiver; if so whether caregiver symptomatic and tested; outcome of caregiver if available,
EHU	Staff interviews	Caregiver accompaniment; identity of caregiver; ability of caregiver to provide care to child (i.e., if too sick to care for child)
ETC	Case investigation form (should travel with patient from EHU)	Date of admission to ETC; location of EHU referred from; date of death or discharge
ETC	Site-specific clinical record	Ebola PCR result; vital signs; symptom progression; receipt of enteral or parenteral fluids; medications given; laboratory results; whether unrelated convalescent patient recruited to care for child
ETC	Admission book	Dates of admission, vital status of child, outcomes in some cases. whether accompanied by caregiver; outcome of caregiver, if available.
WAERC	WAERC database	Cross-referencing and confirmation of demographic data from case investigation forms; confirmation of dates and locations of transfers; cross-referencing of PCR result.
WAERC	Child protection records	Tracing missing outcomes
Burial teams led by Concern Worldwide	Burial records	Tracing missing outcomes
E-Health 117 emergency telephone service	Database of calls received reporting bodies and sick persons	Tracing missing outcomes
Telephone call to head of household		Vital status post discharge, any ongoing health problems (if available)
Survivor clinics	Clinical records of survivors attending clinic	Vital status post discharge, ongoing health problems (if available)
ODCH†	Hospital clinical records	Diagnosis; vital status; laboratory test results if available

*EHU, Ebola holding unit; ETC, Ebola treatment center; ODCH, Ola During Children’s Hospital; WAERC, Western Area Emergency Response Command Centre.
†For children transferred from ETCs with ongoing clinical problems.

Technical Appendix Table 2. Multivariable analysis of 282 Ebola virus–positive children who attended an Ebola holding unit and for whom outcome information was recorded, Western Area, Sierra Leone, August 2014–March 2015*

Variable	Adjusted for all variables with p<0.2 in univariable analysis, OR (95% CI)	Fully adjusted OR (95% CI)
Sex		
F	1	1
M	1.36 (0.80–2.30)	1.44 (0.78–2.65)
Age, y, OR per + 1 y	0.92 (0.86–0.99)	0.94 (0.85–1.04)
Age group, y		
5 to ≤12	1	1
1 to ≤5	1.69 (1.00–2.86)	1.64 (0.77–3.49)
Days from symptom onset to EHU admission, OR per + 1 year	–	0.97 (0.85–1.04)
Common symptoms		
Fever or fatigue	–	1
Fever and fatigue	–	1.00 (0.44–2.27)
Vomiting/nausea		
No	–	1
Yes	–	0.69 (0.28–1.71)
Diarrhea		
No	1	1
Yes	1.84 (1.04–3.25)	1.84 (0.76–4.46)
Anorexia		
No	–	1
Yes	–	1.55 (0.59–4.10)
Abdominal pain		
No	–	1
Yes	–	0.87 (0.40–1.90)
Conjunctivitis		
No	–	1
Yes	–	1.35 (0.60–3.02)
Rare symptoms		
No	–	1
Yes	–	1.81 (0.58–5.60)
Muscle pain		
No	–	1
Yes	–	0.96 (0.23–4.02)
Joint pain		
No	–	1
Yes	–	1.72 (0.43–6.96)
Headache		
No	1	1
Yes	0.56 (0.29–1.07)	0.44 (0.20–1.00)
Difficulty breathing		
No	–	1
Yes	–	2.05 (0.60–7.03)
Difficulty swallowing	–	
No	–	1
Yes	–	0.80 (0.25–2.60)
Admitted accompanied by caregiver		
No	–	1
Yes	–	0.70 (0.19–2.52)
Admission date		
Before Jan 9	1	1
On/after Jan 9	1.84 (0.73–4.62)	3.87 (1.04–14.37)
Common medications		
Antimicrobial or antimalarial drug at EHU	–	1
Antimicrobial and antimalarial drug at EHU	–	0.33 (0.07–1.55)
Intravenous fluids at EHU		
No	–	1
Yes	–	0.35 (0.07–1.70)
EHU		
Ola During Children's Hospital	–	1
Connaught	–	1.13 (0.38–3.33)
Lumley	–	0.36 (0.07–1.69)
Rokupa	–	0.26 (0.08–0.92)
Macauley	–	1.05 (0.24–4.50)
Newton	–	0.50 (0.11–2.36)
Kerry Town	–	0.13 (0.01–1.69)
Police Training Station 2	–	0.19 (0.03–1.12)

Variable	Adjusted for all variables with p<0.2 in univariable analysis, OR (95% CI)	Fully adjusted OR (95% CI)
34 Military Hospital	–	0.31 (0.02–4.46)
Aspen	–	0.15 (0.01–2.37)
Kuntorloh	–	–
Jui	–	0.18 (0.01–2.73)

*OR, odds ratio; –, omission of variables according to p value as described in the analysis and in the column head.

Technical Appendix Table 3. Association between having all symptoms missing (vs. having at least 1 symptom recorded) and key complete variables for 282 Ebola virus–positive children who attended an Ebola holding unit and for whom outcome information was recorded, Western Area, Sierra Leone, August 2014–March 2015 *

Variable	Total	At least 1 symptom recorded	All symptoms missing	p value†
Total	282 (100)	209 (74)	73 (26)	–
Outcome				
Survived	122 (43)	89 (73)	33 (27)	0.697
Died	160 (57)	120 (75)	40 (25)	
Sex				
F	146 (52)	107 (73)	39 (27)	0.743
M	136 (48)	102 (75)	34 (25)	
Age, y				
Mean (± SD)	6.6 (3.9)	6.5 (3.8)	6.8 (4.0)	0.642
Median (interquartile range)	7 (3–10)	6 (3–10)	7 (3–10)	
Admission date				
Before Jan 9	256 (91)	187 (73)	69 (27)	0.199
On/after Jan 9	26 (9)	22 (85)	4 (15)	
Ebola holding unit				
Ola During Children's Hospital	112 (40)	94 (84)	18 (16)	<0.001
Connaught	57 (20)	55 (96)	2 (4)	
Lumley	13 (5)	10 (77)	3 (23)	
Rokupa	24 (9)	8 (33)	16 (67)	
Macauley	16 (6)	5 (31)	11 (69)	
Newton	20 (7)	9 (45)	11 (55)	
Kerry Town	5 (2)	3 (60)	2 (40)	
Police Training Station 2	14 (5)	13 (93)	1 (7)	
34 Military Hospital	10 (4)	2 (20)	8 (80)	
Aspen‡	4 (1)	–	–	
Kuntorloh‡	1 (0)	–	–	
Jui	6 (2)	5 (83)	1 (17)	

*All values are no. (%) unless indicated otherwise. –, dropped from the analysis because of insufficient numbers in all cells.
†χ^2 testing overall association of variable with missing symptom status variable.
‡Dropped from χ^2 analysis because of insufficient numbers in all cells.

Technical Appendix Table 4. Comparison of the imputed study dataset with a complete records approach for 282 Ebola virus–positive children who attended an EHU and for whom outcome information was recorded, Western Area, Sierra Leone, August 2014–March 2015*

Variable	Crude OR (95% CI)	
	Complete records†	Multiple imputation‡
Sex		
F	1	1
M	1.49 (0.93–2.39)	1.44 (0.78–2.65)
Age, y, OR per + 1 y	0.91 (0.85–0.97)	0.94 (0.85–1.04)
Age group, y		
5 to ≤12	1	1
1 to ≤5	1.88 (1.14–3.11)	1.88 (1.14–3.11)
Days from symptom onset to EHU admission, OR per +1 d	0.99 (0.90–1.08)	1.16 (0.59–2.29)
Common symptoms		
Fever or fatigue	1	1
Fever and fatigue	0.85 (0.20–3.68)	1.07 (0.58–1.97)
Vomiting/nausea		
No	1	1
Yes	1.12 (0.64–1.97)	1.13 (0.64–2.00)
Diarrhea		
No	1	1
Yes	2.05 (1.15–3.65)	1.94 (1.11–3.39)
Anorexia		
No	1	1
Yes	1.33 (0.68–2.60)	1.30 (0.66–2.55)
Abdominal pain		
No	1	1
Yes	0.80 (0.45–1.45)	0.82 (0.49–1.40)
Difficulty swallowing		
No	1	1
Yes	1.01 (0.51–2.00)	0.98 (0.44–2.17)
Difficulty breathing		
No	1	1
Yes	1.76 (0.72–4.28)	1.74 (0.69–4.55)
Muscle pain		
No	1	1
Yes	0.91 (0.51–1.63)	0.93 (0.53–1.64)
Joint pain		
No	1	1
Yes	0.98 (0.55–1.76)	1.00 (0.57–1.77)
Headache		
No	1	1
Yes	0.63 (0.35–1.14)	0.64 (0.34–1.19)
Conjunctivitis		
No	1	1
Yes	1.18 (0.65–2.14)	1.10 (0.63–1.90)
Rare symptoms		
No	1	1
Yes	1.48 (0.56–3.90)	2.04 (0.84–4.97)
Admitted accompanied by caregiver		
No	1	1
Yes	1.34 (0.73–2.45)	1.22 (0.65–2.28)
Date of admission		
Before Jan 9	1	1
On/after Jan 9	1.81 (0.76–4.31)	1.81 (0.76–4.31)
Common medications		
Antimicrobial or antimalarial drugs at EHU or ETC	1	1
Antimicrobial and antimalarial drugs at EHU or ETC	0.65 (0.25–1.71)	0.64 (0.32–1.30)
Intravenous fluids at EHU		
No	1	1
Yes	1.07 (0.33–3.46)	1.07 (0.33–3.46)
EHU		
Ola During Children's Hospital	1	1
Connaught	1.07 (0.55–2.08)	1.06 (0.55–2.08)
Lumley	0.49 (0.16–1.57)	0.49 (0.16–1.57)
Rokupa	0.35 (0.14–0.86)	0.35 (0.14–0.86)
Macauley	1.27 (0.41–3.91)	1.27 (0.41–3.91)
Newton	0.58 (0.22–1.50)	0.58 (0.22–1.50)
Kerry Town	0.38 (0.06–2.40)	0.38 (0.06–2.40)
Police Training School 2	0.32 (0.10–1.02)	0.32 (0.10–1.02)

Variable	Crude OR (95% CI) Complete records†	Multiple imputation‡
34 Military Hospital	0.58 (0.16–2.11)	0.58 (0.16–2.11)
Aspen	0.58 (0.08–4.26)	0.58 (0.08–4.26)
Jui	0.29 (0.05–1.65)	0.29 (0.05–1.65)

*EHU, Ebola holding unit; ETC, Ebeol treatment center; OR, odds ratio
†Complete records: only patients with complete records for the variable in question were analyzed.
‡Multiple imputation used to account for missing data, with variables included in the multiple imputation model in accordance with Table 2.

Technical Appendix Table 5. Descriptive, univariable, and multivariable analysis of 193 Ebola virus–positive children who entered an EHU, who survived to attend an ETC, and for whom outcome information was recorded, Western Area, Sierra Leone, August 2014–March 2015*

Variable	Total	Survived	Died	Crude OR (95% CI)†	Multivariable adjusted OR‡
Total§	193 (100)	119 (62)	74 (38)	–	–
Had diarrhea at EHU					
No	83 (43)	55 (66)	28 (34)	1	–
Yes	52 (27)	29 (56)	23 (44)	1.47 (0.72–3.0)	–
Days between EHU admission and ETC transfer					
Mean (± SD)	2.6 (2.1)	2.7 (2.1)	2.6 (2.2)	1.00 (0.86–1.14)	–
Median (IQR)	2 (2–3)	2 (2–3)	2 (1–3)	–	–
Transfer distance, km					
Mean (± SD)	52.9 (85.5)	46.3 (76.6)	63.3 (96.9)	1.00 (0.99–1.01)	–
Median (IQR)	20 (14–45)	25 (18–42)	20 (14–45)	*OR for each +1 km*	–
Transfer distance, km, binary					
0	26 (13)	17 (65)	9 (35)	1	–
>0	167 (87)	102 (61)	65 (39)	1.20 (0.51–2.86)	–
Transfer distance, km, 4 groups					
0	26 (13)	17 (65)	9 (35)	1	–
1 to <20	38 (20)	22 (58)	16 (42)	1.37 (0.49–3.86)	–
20 to <50	103 (53)	68 (66)	35 (34)	0.97 (0.39–2.40)	–
≥50	26 (14)	12 (46)	14 (54)	2.20 (0.72–6.73)	–
ETC					
Kerry Town confirmed beds (1)	55 (28)	39 (71)	16 (29)	1	1
Police Training School 1 (2)	67 (35)	48 (72)	19 (28)	0.96 (0.44–2.12)	1.07 (0.48–2.40)
Police Training School 2 (3)	14 (7)	9 (64)	5 (36)	1.35 (0.39–4.67)	1.20 (0.34–4.22)
34 Military Hospital (4)	7 (4)	3 (43)	4 (57)	3.25 (0.65–16.20)	2.92 (0.57–15.02)
MSF POW (5)	11 (6)	2 (18)	9 (82)	10.97 (2.13–56.49)	13.30 (2.50–70.71)
Bo (6)	9 (5)	4 (44)	5 (56)	3.05 (0.72–12.83)	3.45 (0.80–14.89)
Kenema (7)	14 (7)	6 (43)	8 (57)	3.25 (0.97–10.88)	3.72 (1.08–12.81)
Aspen (8)	11 (6)	6 (55)	5 (45)	2.03 (0.54–7.62)	2.25 (0.59–8.60)
Kailahun (9)	1 (1)	1 (100)	0	–	–
Adventist Development and Relief Agency (10)	1 (1)	1 (100)	0	–	–
Goderich (11)	3 (2)	0	3 (100)	–	–
Medication received					
Had antimicrobial drug at EHU or ETC¶					
No	5 (3)	2 (40)	3 (60)	1	–
Yes	124 (65)	75 (60)	49 (40)	0.78 (0.18–3.39)	–
Had antimalarial drug at EHU or ETC					
No	27 (14)	19 (70)	8 (30)	1	–
Yes	102 (53)	75 (60)	49 (40)	1.60 (0.71–3.63)	–
Had antiemetic drug at EHU or ETC					
No	91 (47)	53 (58)	38 (42)	1	–
Yes	38 (20)	24 (63)	14 (37)	0.92 (0.45–1.89)	–
Had intravenous fluids at ETC					
No	148 (77)	93 (62)	55 (38)	1	–
Yes	45 (23)	26 (58)	19 (42)	1.24 (0.63–2.44)	–

*All results are no. (%), unless otherwise indicated. EHU, Ebola holding unit; ETC, Ebola treatment center; IQR, interquartile range; MSF POW, Médecins Sans Frontières Prince of Wales; OR, odds ratio; –, variables omitted because p>0.2 as described in the notes or p<0.1 as described in the notes.
†OR (95% CI), with multiple imputation used to account for missing data. Multiple imputation model comprised antimicrobial drug received, antiemetic drug received, antimalarial drug received, presence of diarrhea, whether admitted to EHU with caregiver, time from symptom onset to admission, days between admission to EHU and transfer to ETC, date of admission, specific EHU, specific ETC, sex, outcome status, distance between EHU and ETC, and age.
‡Variables selected for inclusion in analysis using a backward stepwise approach (first selecting variables with p<0.2 in univariable analysis, before removing those with p<0.1 one by one). Multiple imputation applied in accordance with †.
§Total children admitted to ETC with complete outcome information.

Technical Appendix Table 6. Multivariable analysis of 193 Ebola virus–positive children who entered an EHU and survived to attend an ETC and for whom outcome information was recorded (alternative models), Western Area, Sierra Leone, August 2014–March 2015*

Variable	Adjusted for all variables with p<0.2 in univariable analysis, OR (95% CI)	Fully adjusted OR (95% CI)
Had diarrhea at EHU		
No	–	1
Yes	–	1.16 (0.54–2.47)
Days between EHU admission and ETC transfer		
Mean (± SD)	–	1.00 (0.84–1.20)
Median (IQR)		–
Transfer distance, km		
Mean (± SD)	1.03 (0.99–1.07)	1.01 (0.97–1.05)
Median (IQR)	OR for each +1 km	OR for each +1 km
ETC		
Kerry Town confirmed beds	1	1
Police Training School 1	2.33 (0.70–7.73)	1.62 (0.41–6.43)
Police Training School 2	2.69 (0.38–19.22)	2.38 (0.35–16.28)
34 Military Hospital	8.24 (0.92–73.71)	5.57 (0.59–52.26)
Médecins Sans Frontières Prince of Wales	21.63 (2.69–173.97)	16.57 (1.99–138.14)
Bo	–	–
Kenema	–	–
Aspen	4.18 (0.68–25.6)	3.00 (0.46–19.69)
Kailahun	–	–
Adventist Development and Relief	–	–
Goderich	–	–
Had antimicrobial drug at EHU or ETC		
No	–	1
Yes	–	0.77 (0.13–4.56)
Had antimalarial drug at EHU or ETC		
No	–	1
Yes	–	1.25 (0.39–4.10)
Had antiemetic drug at EHU or ETC		
No	–	1
Yes	–	1.04 (0.40–2.71)
Had intravenous fluids at ETC		
No	–	1
Yes	–	1.74 (0.62– 4.88)

*EHU, Ebola holding unit; ETC, Ebola treatment center; IQR, interquartile range; OR, odds ratio; –, omission of variables according to p value as described in the analysis.

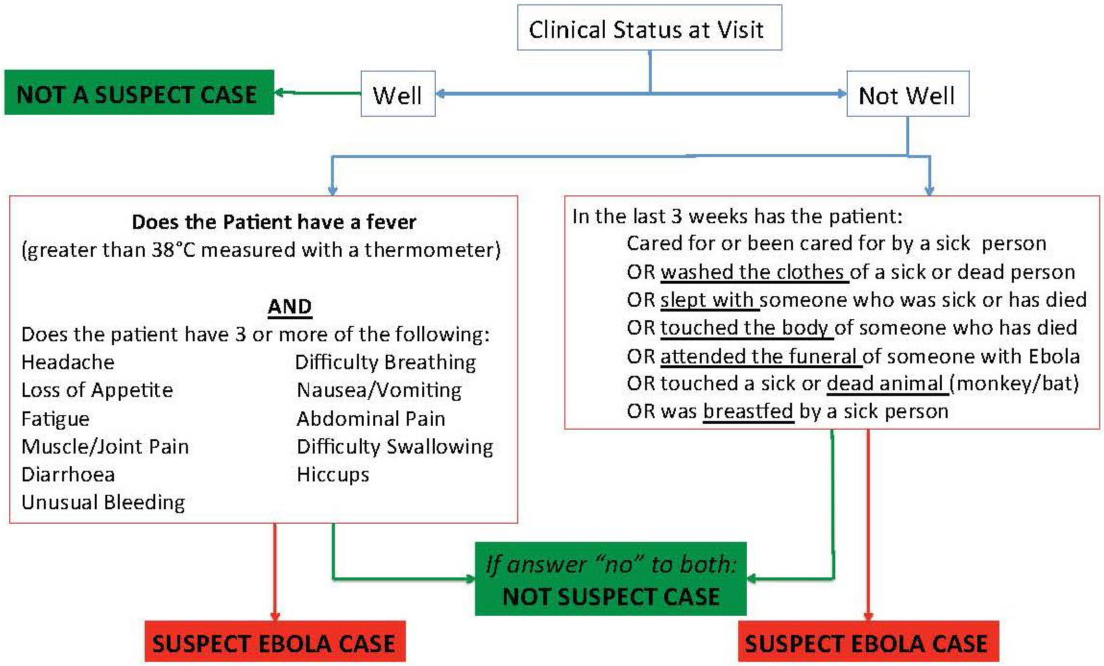

The symptom checklist is modified for children as below:

**Children <5 years**	**Children ≥5 years**
Fever or history of fever within 48 hours	Fever or history of fever within 48 hours
Vomiting	Vomiting or nausea
Appetite loss	Appetite loss
Diarrhoea	Diarrhoea
Difficulty breathing	Difficulty breathing or swallowing
Excessive crying	Headache
Unexplained bleeding (nose, gums, gastrointestinal or other)	Unexplained bleeding (nose, gums, gastrointestinal or other)
Red eyes and or rash	Red eyes and or rash
Prostration	Weakness or severe fatigue
	Generalised muscular or articular pain
**If fever (or history of fever) and ≥1 symptom isolate child**	**If fever (or history of fever) and ≥2 symptoms isolate child**

Technical Appendix Figure. Screening flowchart for Ebola virus disease used on attendance at healthcare facilities to classify patients as "suspect Ebola case" or "not suspect case," Western Area, Sierra Leone (*2*).
